# Vitamin C Lowers Blood Pressure in Spontaneously Hypertensive Rats by Targeting Angiotensin-Converting Enzyme I Production in a Frequency-Dependent Manner

**DOI:** 10.1155/2022/9095857

**Published:** 2022-07-08

**Authors:** Eun-Sang Hwang, Ga-Young Choi, Kwan Joong Kim, Min-Jeong Kim, Seok Lee, Jin-Won Lee, Dae-Ok Kim, Ji-Ho Park

**Affiliations:** ^1^Department of Gerontology (Age Tech-Service Convergence Major), Graduate School of East-West Medical Science, Kyung Hee University, Yongin 17104, Republic of Korea; ^2^Department of East-West Medicine, Graduate School of East-West Medical Science, Kyung Hee University, Yongin 17104, Republic of Korea; ^3^Graduate School of Biotechnology, Kyung Hee University, Yongin 17104, Republic of Korea; ^4^Department of Food Science and Biotechnology, Kyung Hee University, Yongin 17104, Republic of Korea

## Abstract

The lowering blood pressure effect of vitamin C (VC) has been evaluated in various models. As VC has a fast degradation rate in the body after consumption, a study of the frequency-dependent manner of VC is essential for the sustained antihypertension effect of VC. In this study, we investigated the frequency and dose dependency of vitamin C (VC) on blood pressure reduction in spontaneously hypertensive rats (SHRs). Wistar–Kyoto rats (WKYs) and SHRs were orally administered tap water or VC (250, 500, 1000, and 2000 mg/60 kg/day). Blood pressures were measured using the tail-cuff method, and thoracic aortas, liver, and blood were harvested from sacrificed rats after 8 weeks to measure angiotensinogen, angiotensin-converting enzyme (ACE) I, endothelial nitric oxide synthase (eNOS), and total nitric oxide (NOx) concentration. VC decreased blood pressure from the fourth week with no significant differences between doses. The twice-a-day administration of VC decreased blood pressure from the second week, and the blood pressure in these groups was close to that of the WKY group in the eighth week. Treatment with once a day VC decreased ACE I production which was further significantly reduced in twice a day groups. Angiotensinogen and eNOS production were increased upon VC treatment but were not significant among groups. The NOx content was decreased by VC treatment. These results suggest that VC lowers blood pressure in SHRs by directly targeting ACE I production in a frequency-dependent manner and may improve endothelial function depending on the frequency of administration.

## 1. Introduction

Essential hypertension, in which the cause of high blood pressure has not been clearly identified, is the most common type of hypertension, affecting 85% of individuals with high blood pressure [1]. Essential hypertension frequently clusters with other risk factors such as aging, diabetes, and hyperlipidemia to cause target organ damage, which induces catastrophic events such as stroke, heart attack, and renal failure [2].

The renin-angiotensin system (RAS) is one of the main mechanisms for regulating blood pressure, hence defects in this system are thought to drive the pathophysiology of hypertension [3]. In RAS, production of angiotensinogen in the liver is cleaved by renin to form angiotensin (Ang) I. Then, angiotensin-converting enzyme (ACE) I converts Ang I to Ang II, which leads to contraction of the muscles surrounding blood vessels and increase in aldosterone level, blood pressure, and superoxide production, resulting in impaired endothelial functions [3–5]. Although ACE I inhibitors, such as captopril and ramipril, are commonly used medications for patients with hypertension, they cause adverse side effects such as cough, hyperkalemia, and dizziness [6]. Therefore, diet-related lifestyle modifications have been emphasized to effectively prevent and control high blood pressure [7–9].

Previous studies have shown that the pathogenesis of hypertension is accompanied by endothelial dysfunction with intravascular oxidative stress and loss of nitric oxide (NO) availability [10–13]. NO is mainly produced from L-arginine by the endothelial nitric oxide synthase (eNOS) and plays a major role in the regulation of vascular tone by inducing relaxation of vascular smooth muscle [14]. Due to antioxidants' ability to prevent oxidative stress and ameliorate vascular endothelial dysfunction [15], many antioxidants have been proven to be beneficial in regards to cardiovascular disease [16–19].

Vitamin C (ascorbic acid; VC) is a water-soluble vitamin that is naturally present in fruit and vegetables. Unlike most animals, humans cannot synthesize VC, making it an essential dietary component [20]. According to National Institutes of Health researchers, repeated doses of VC as low as 200 mg per day saturate the human body. Moreover, high doses of VC are more rapidly excreted from the body. In this case, VC has a half-life of about 30 min [21–23]. King et al. [24] discovered that 500 mg of VC taken orally every 12 hrs is sufficient to provide continuous excretion of excess VC into the urine, but high dose consumption once a day caused detectable excretion from about 4 to 16 hrs later. VC is a potent antioxidant that reduces oxidative stress and enhances endothelial function by scavenging intracellular superoxide, thereby activating smooth muscle guanylyl cyclase and eNOS, which leads to the production of NO [25]. The antihypertensive effect of VC has been evaluated in animal models and human studies [9, 26–29]. Although most hypertensive patients exhibit essential hypertension, salt or inflammatory agents were used to induce hypertension in the animal models [27, 28]. Further, VC has a fast degradation rate in the body, and its effect is dependent on its physiological concentration [21, 25]. However, the frequency-dependent effect of VC administration on hypertension has not been verified.

In this study, we investigated the effects of dose dependency and frequency dependency of VC on blood pressure in a spontaneously hypertensive rat (SHR). Furthermore, we used Western blot analysis and the Griess test to elucidate the target of VC in the RAS and eNOS-NO pathway that was responsible for its frequent effect on hypertension.

## 2. Materials and Methods

### 2.1. Materials

VC was obtained from Kwang Dong Pharmaceutical Co., Ltd. (Seoul, South Korea). Bovine serum albumin (V900933), sodium dodecyl sulfate (SDS) (L3771), ammonium persulfate (A3678), skim milk powder (70166), polysorbate 20 (Tween 20) (P9416), Tris-base (10708976001), Tris-HCl (10812846001), and glycine (G7126) were purchased from Sigma–Aldrich (St. Louis, MO, USA).

### 2.2. Animals

Thirty-five-week-old male SHRs (approximately 200 g each) and five age-matched male Wistar–Kyoto rats (WKYs) were purchased from Oriental Bio Inc. (Sungnam, South Korea). Rats were housed in a laboratory cage under controlled conditions: a 12/12 hrs (lights on at 08:00 a.m. and off at 8:00 p.m.) light/dark cycle, 50 ± 5% of humidity, and 25 ± 1°C of indoor temperature. Each cage accommodated two of rats for sufficient space. Rats had access to a standard diet (5L79; Orient Bio Inc.) and water ad libitum throughout the experiment period. All animal procedures complied with the Institutional Care and Use Committee of Kyung Hee University with approval number: KHUASP (SE)-17-019 (approval date: 12 June 2017) and were performed in accordance with the guiding principles for the care and use of animals approved by the Council of the National Institutes of Health Guide for the Care and Use of Laboratory Animals.

### 2.3. Oral Administration of VC

**Figure 1:**
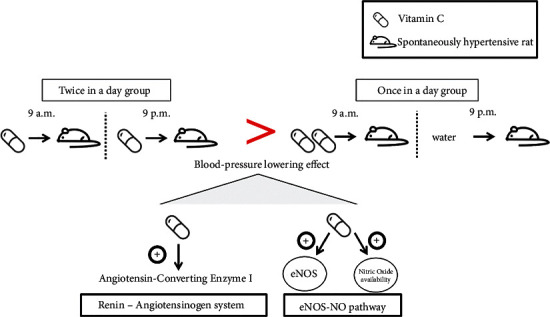
Summary of vitamin C (VC) administration and mechanisms associated with the blood-pressure-lowering action of VC. The dose-dependent groups were administered VC 250, VC 500, VC 1000, and VC 2000 (units in mg/60 kg/day) orally at 9 a.m. and tap water at 9 p.m. each day for 8 weeks. The frequency-dependent groups were administered with VC 125 *∗* 2 and VC 250 *∗* 2 (units in mg/60 kg body weight/day) at 9 a.m. and 9 p.m. twice a day for 8 weeks. Systolic blood pressure (SBP) and diastolic blood pressure (DBP) were assessed by a noninvasive tail-cuff method once every two weeks at the same time of the day. The target of VC in the renin-angiotensin system (RAS) and endothelial nitric oxide synthase (eNOS)-nitric oxide (NO) pathway responsible for its frequent effect on hypertension was elucidated through Western blot analysis and the Griess test.

After one week of adaptation, SHRs were randomly divided into seven groups, consisting of five rats per group. These seven groups were each randomly assigned to a SHR group and six VC-treated groups. The WKY group (normotensive group) and SHR group (control group) were orally administered tap water each day for 8 weeks at 9 a.m. and 9 p.m. twice in a day. SHR groups with VC 250, VC 500, VC 1000, and VC 2000 (units in mg/60 kg body weight/day) were orally administered VC at 9 a.m. and tap water at 9 p.m. each day for 8 weeks. SHRs with VC 125 *∗* 2 and VC 250 *∗* 2 groups (units in mg/60 kg body weight/day) were orally administered each day for 8 weeks at 9 a.m. and 9 p.m. twice in a day (Figure 1).

### 2.4. Measurement of Blood Pressure

Systolic blood pressure (SBP) and diastolic blood pressure (DBP) were measured by a noninvasive tail-cuff method using CODA™ tail-cuff blood pressure system (Kent Scientific Corp., Torrington, CT, USA) once every two weeks at the same time of the day. The rats were kept at 37°C for 15 min in a black acryl animal holder before measuring the blood pressure to intensify the pulsation of the tail artery and minimize stress.

### 2.5. Collection of Tissue and Serum

After 8 weeks of oral administration of tap water and VC, the experimental animals were sacrificed. The animals were anesthetized with ethyl ether, and then their liver and thoracic aorta were rapidly harvested. Blood was collected in a serum-separating tube (BD Vacutainer™ SST™ II Advance Tubes; Thermo Fisher Scientific, Waltham, MA, USA) with an anticoagulant and centrifuged at 18,033 × *g* 4°C for 20 min (PK121R; ALC International S.R.L., Cologno Monzese, Italy). Aliquots of serum were stored at −80°C prior to analysis.

### 2.6. Western Blot

The livers and arteries were homogenized with lysis buffer. The homogenized tissues in the lysis buffer were sonicated (NRE-02; Next Advance, Troy, NY, USA), centrifuged at 18,033 × *g* 4°C for 20 min, and the supernatant was used for biochemistry assays. The concentration of proteins was quantitated by the Bradford protein assay. The samples were separated using 10% SDS-PAGE, transferred to polyvinylidene fluoride membranes, and blocked with 5% skim milk dissolved in Tris-buffered saline containing 0.1% Tween 20 for 1 hr at room temperature. Then, the membrane was incubated with antiangiotensinogen (ab213705, dilution 1 : 1000; Abcam plc, Cambridge, UK), anti-ACE I (ab11734, dilution 1 : 1000; Abcam plc), anti-eNOS (ab76198, dilution 1 : 1000; Abcam plc), and anti-*β*-actin (ab20272, dilution 1 : 1000; Abcam plc) primary antibodies overnight at 4°C, followed by horseradish peroxidase conjugated goat anti-mouse (ab205719, dilution 1 : 1000; Abcam plc) and goat anti-rabbit (ab205718, dilution 1 : 1000; Abcam plc) secondary antibodies were treated and reacted at room temperature for 1 hr. After the reaction, the membrane was visualized with ECL solution (EzWestLumi plus; ATTO, Amherst, NY, USA) which reacts with horseradish peroxidase and was analyzed using Image J software (version 1.49; Bethesda, MD, USA).

### 2.7. Total NO Measurement

The NOx (total nitric oxide metabolites) was measured using a commercial kit (23479; Sigma–Aldrich) according to the manufacturer's instructions. Nitrate in serum was reduced by nitrate reductase to nitrite and determined after Griess reaction. Absorbance of reacted samples was measured by a microplate reader (Victor X3; PerkinElmer, Waltham, MA, USA) at 540 nm, and NOx was determined using a nitrite standard calibration curve.

### 2.8. Statistical Analysis

The results were expressed as the mean ± standard error of the mean. Statistical comparisons were conducted using repeat-measure, one-way analysis of variance followed by Tukey's honestly significant difference (HSD) test using SPSS 25.0 (SPSS Inc., Chicago, IL, USA). Differences were considered as statistically significant at *p* < 0.05.

## 3. Results

### 3.1. Effect of Oral Administration Dose of VC on Blood Pressure in SHRs

**Figure 2:**
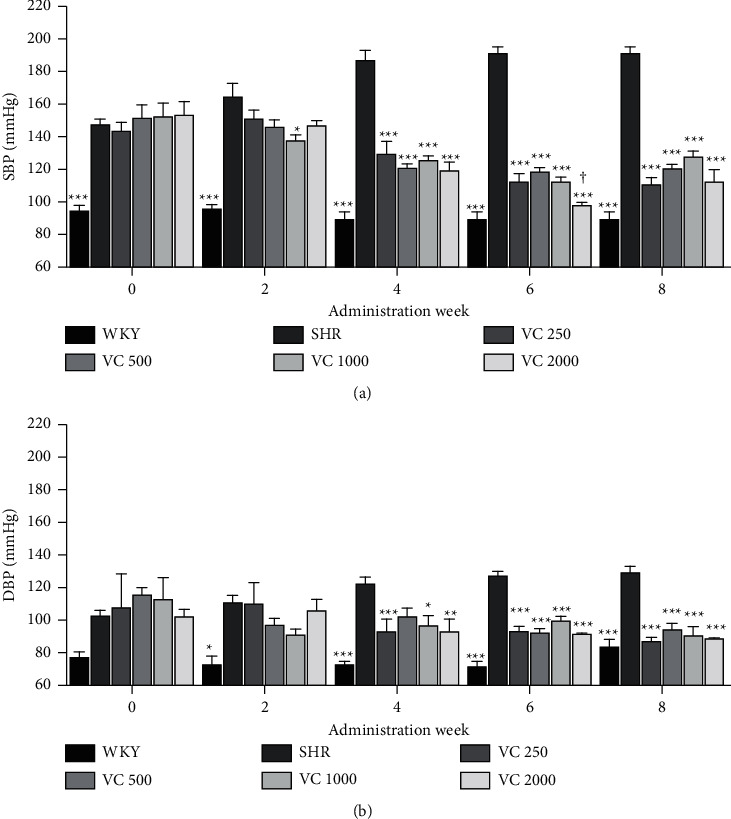
Blood pressure of the Wistar–Kyoto rats (WKYs), spontaneously hypertensive rats (SHRs), and SHRs with the dose of vitamin C (VC) administration. (a) Systolic blood pressure (SBP) and (b) diastolic blood pressure (DBP) of the WKY group (a normotensive group; tap water at 9 a.m. and 9 p.m.), SHR group (a control group; tap water at 9 a.m. and 9 p.m.), and VC-administered SHR groups (250, 500, 1000, and 2000 mg/60 kg body weight/day of VC; VC at 9 a.m. and tap water at 9 p.m.) over 8 weeks. Each group consists of five rats. Tukey's honestly significant difference (HSD) test: ^*∗*^*p* < 0.05, ^*∗*^^*∗*^*p* < 0.01, ^*∗∗∗*^*p* < 0.001 vs. SHR group for each week, ^†^*p* < 0.05 vs. VC 500 group for each week.

The SBP and DBP of the WKY group, SHR group, and VC 250, 500, 1000, and 2000 groups for 8 weeks are shown in Figure 1. The SBP of the WKY group (94.20 ± 3.60 mmHg) was lower than that of the SHR group (146.27 ± 4.50 mmHg; *p* < 0.001), and there were no significant differences in SBP between the SHR group and VC groups at the beginning (0 week) of the study (Figure 2(a)). The SBP of the WKY group was not significantly changed during the experimental period, whereas the SBP of the SHR group was gradually and significantly increased in comparison with 0 week over the experimental period (second week: 164.49 ± 7.78 mmHg, *p*=0.178; fourth week: 186.62 ± 6.06 mmHg, *p* < 0.001; sixth week: 191.09 ± 3.96 mmHg, *p* < 0.001; eighth week: 191.12 ± 4.54 mmHg, *p* < 0.001 vs. 0 week; Figure S1(a)). In the second week, the SBP of the VC groups was not significantly different compared to the SHR group (VC 250: *p*=0.369, VC 500: *p*=0.127, VC 2000: *p*=0.151 vs. SHR group) except for the VC 1000 group (*p*=0.011), but after the fourth week, the SBPs were decreased in all administrations of VC groups compared with the SHR group, and there was no significant difference between the VC groups except for the between VC 500 and VC 2000 groups (*p*=0.013) in the sixth week (Figure 2(a)).

The DBP between the WKY group (76.17 ± 3.85 mmHg) and the SHR group (101.32 ± 4.39 mmHg) was not significantly different (*p*=0.581) at the beginning (0 week) of the study, but after the second week, the DBP of the WKY group was significantly lower than the SHR group (Figure 2(b)). Like as SBP, DBP of the WKY group was not significantly changed during the experimental period, whereas DBP of the SHR group was gradually increased over the experimental period (second week: 109.08 ± 5.21 mmHg, fourth week: 121.59 ± 4.14 mmHg, sixth week: 126.32 ± 3.17 mmHg, eighth week: 128.50 ± 4.18 mmHg; Figure S1(b)). VC administration decreased DBP at VC 250, VC 1000, and VC 2000 groups in the fourth week (*p*=0.009, 0.22, and 0.008, respectively), and after sixth week, DBPs were significantly decreased by VC administration compared with the SHR group (*p* < 0.001, all VC-administered groups in the sixth and eighth week; Figure 2(b)).

### 3.2. Effect of Oral Administration Frequency of VC on Blood Pressure in SHRs

**Figure 3:**
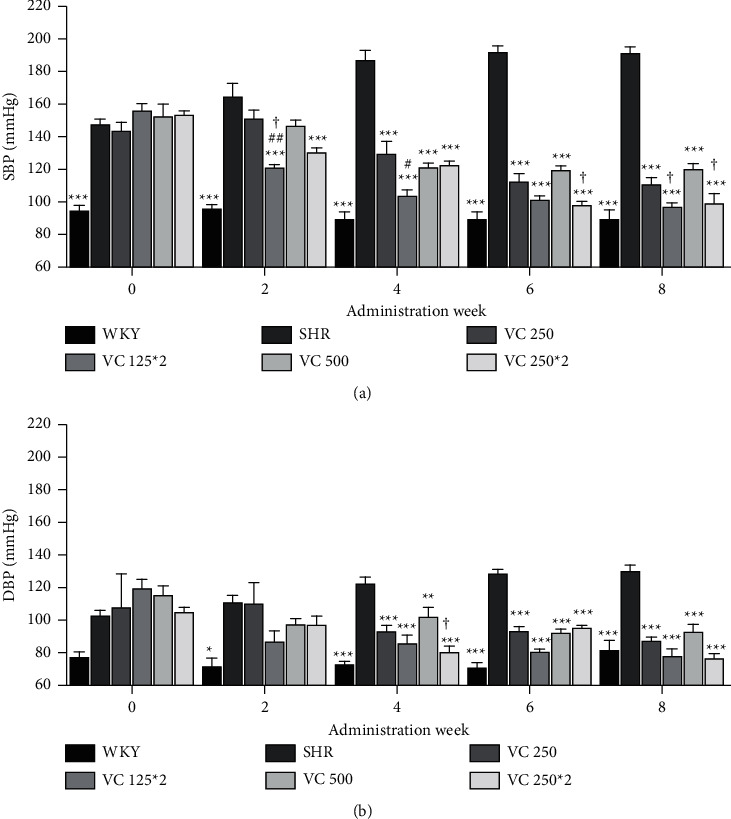
Effects of the frequency of vitamin C (VC) administration on blood pressure of the Wistar–Kyoto rats (WKYs) and spontaneously hypertensive rats (SHRs). (a) Systolic blood pressure (SBP) and (b) diastolic blood pressure (DBP) of the WKY group (a normotensive group; tap water at 9 a.m. and 9 p.m.), SHR group (a control group; tap water at 9 a.m. and 9 p.m.), and VC-administered SHR groups (250 and 500 mg/60 kg body weight/day of VC; VC at 9 a.m. and tap water at 9 p.m.; 125*∗*2 and 250*∗*2 mg/60 kg body weight/day of VC; VC at 9 a.m. and 9 p.m.) over 8 weeks. Each group consists of five rats. Tukey's HSD test: ^*∗*^*p* < 0.05, ^*∗∗*^*p* < 0.01, vs. SHR group for each week, ^#^*p* < 0.05, ^##^*p* < 0.01 vs. VC 200 group for each week, vs. VC 500 group for each week.

We evaluated changes of SBP and DBP in SHRs according to the administration frequency of VC. As the VC-administrated groups did not show significant differences, we chose two low-dose groups (VC 250 and VC 500) to compare the lowering blood pressure effect in a frequency-dependent manner. In the second week, SBP of two groups, which were treated with VC twice a day (VC 125 *∗* 2 and VC 250 *∗* 2 groups), showed significant decreases compared with the SHR group (*p* < 0.001; both of them) as well as the 0-week SBP (*p* < 0.001 and *p*=0.007, respectively) of each group (Figures 3(a) and S2(a)). However, VC once a day groups (VC 250 and VC 500 groups) were not significant compared with SHR groups, although the total dose of VC on one day was the same between once a day groups and twice a day groups (Figures 3(a) and S2(a)). In addition, the VC 125 *∗* 2 group exhibited a greater reduction in SBP than the VC 250 (*p*=0.003) and VC 500 (*p*=0.015) groups in the second week (Figure 3(a)). Moreover, SBPs of the VC 125 *∗* 2 and VC 250 *∗* 2 groups showed a gradual decrease and eventually matching to that of the WKY group in the sixth week (*p*=0.396 and 856, respectively) and eighth week (*p*=0.849 and 0.677, respectively) but not VC 250 and VC 500 groups (sixth week, *p*=0.008 and 0.001; eighth week, *p*=0.039 and 0.002; data not shown).

In the second week, DBP showed no significant difference between the SHR group and VC-administered SHR groups (Figure 3(b)). After fourth week, all VC-administered groups showed significant decreases of DBP compared with the SHR group (Figure 3(b)). And, SHR groups treated with VC twice a day (VC 125 *∗* 2 and VC 250 *∗* 2 groups) showed lower DBP than SHR groups treated with VC once a day (VC 250 and VC 500 groups) in the fourth week and eighth week, but the differences were not significant except for the VC 250 *∗* 2 group (*p*=0.010 vs. VC 500 group) in the fourth week (Figure 3(b)).

### 3.3. Effect of VC on Angiotensinogen and ACE I Protein Production in Liver Tissue

**Figure 4:**
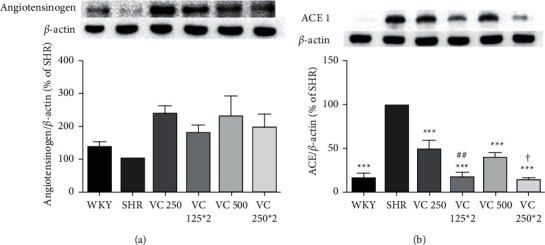
Effects of frequency of vitamin C (VC) administration on (a) angiotensinogen (50 kDa) and (b) angiotensin-converting enzyme (ACE) I (150 kDa) protein expression in the liver of the Wistar–Kyoto rat (WKY) and spontaneously hypertensive rat (SHR) after 8 weeks with representative image. WKY group (a normotensive group; tap water at 9 a.m. and 9 p.m.), SHR group (a control group; tap water at 9 a.m. and 9 p.m.), and VC- administered SHR groups (250 and 500 mg/60 kg body weight/day of VC; VC at 9 a.m. and tap water at 9 p.m.; 125^*∗*^2 and 250^*∗*^2 mg/60 kg body weight/day of VC; VC at 9 a.m. and 9 p.m.) after 8 weeks of administration. Each group consists of five rats. Tukey's HSD test: ^*∗∗∗*^*p* < 0.001 vs. SHR group, ^##^*p* < 0.01 vs. VC 250 group, vs. VC 500 group.

We evaluated angiotensinogen and ACE I protein production in the liver tissue after 8 weeks of blood pressure measurements (Figure 4). Angiotensinogen protein was increased in the VC 250 (241.39 ± 23.32%), 125 *∗* 2 (180.89 ± 23.54%), 500 (231.56 ± 62.55%), and 250 *∗* 2 (201.18 ± 36.80%) SHR groups compared with the SHR group but did not significantly differ between groups (Figure 4(b)).

All VC-administered groups exhibited decreased production of ACE I protein in the liver tissue compared with the SHR group (*p* < 0.001). Moreover, VC 125 *∗* 2 (18.05 ± 5.24%) and VC 250 *∗* 2 (14.86 ± 3.27%) groups showed significant decreased ACE I production compared with the VC 250 (48.92 ± 11.47%) and VC 500 (40.22 ± 6.03%) groups (*p*=0.019 vs. VC 250 group, *p*=0.032 vs. VC 500 group; Figure 4(b)).

### 3.4. Effect of VC on eNOS Production in Aorta Tissue and NO Concentration in Blood Serum in SHRs

**Figure 5:**
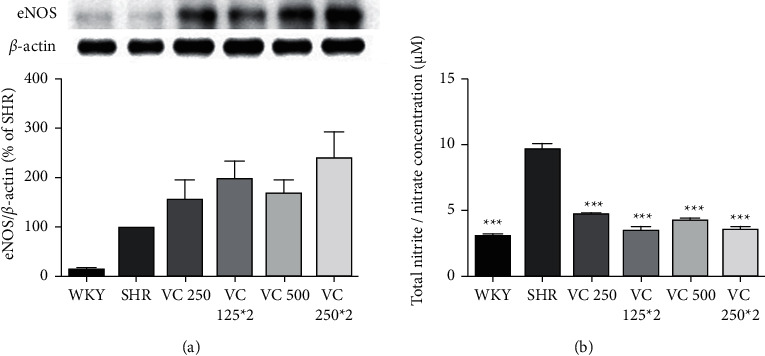
Effects of frequency of vitamin C (VC) administration on (a) endothelial nitric oxide synthase (eNOS) (133 kDa) production in the aorta tissue with representative image and (b) total nitric oxide (NOx) concentration of blood serum in the Wistar–Kyoto rat (WKY) and spontaneously hypertensive rat (SHR) after 8 weeks of blood pressure measurements. WKY group (a normotensive group; tap water at 9 a.m. and 9 p.m.), SHR group (a control group; tap water at 9 a.m. and 9 p.m.), and VC-administered SHR groups (250 and 500 mg/60 kg body weight/day of VC; VC at 9 a.m. and tap water at 9 p.m.; 125 *∗* 2 and 250 *∗*2 mg/60 kg body weight/day of VC; VC at 9 a.m. and 9 p.m.) after 8 weeks of administration. Each group consists of five rats. Tukey's HSD test: ^*∗∗∗*^*p* < 0.001 vs. SHR group.

eNOS production in the WKY group (14.80 ± 3.21%) was lower than that in the SHR group. Although there was no statistically significant difference, the VC 250 (156.13 ± 41.50%), VC 125 *∗* 2 (201.36 ± 32.94%), VC 500 (172.68 ± 25.02%), and VC 250 *∗* 2 (240.27 ± 53.08%) SHR groups showed higher eNOS production than the SHR group (Figure 5(a)).

The total NOx concentration in the blood serum of WKYs and SHRs was measured using a Griess kit. The NOx concentration in the WKY (3.32 ± 0.22 *μ*M) group was significantly lower than that in the SHR (10.41 ± 0.42 *μ*M) group (*p* < 0.001). Further, the VC 250 (5.10 ± 0.17 *μ*M), VC 125 *∗* 2 (3.77 ± 0.45 *μ*M), VC 500 (4.71 ± 0.17 *μ*M), and VC 250 *∗* 2 (3.96 ± 0.32 *μ*M) groups had significant decreased in NOx concentration in the blood serum compared with the SHR group (*p* < 0.001). There was no significant difference in NOx level among the VC-treated SHR groups (Figure 5(b)).

## 4. Discussion

In this study, we evaluated the effect of VC administration on blood pressure as well as components of the RAS and eNOS-NO pathway in SHRs. The SHR is a well-established model for human essential hypertension. An overall change in the RAS components that increased oxidative stress, which produces high blood pressure, has been observed in the SHR model [7, 8, 30–32]. A variety of antioxidants have been shown to produce antihypertensive effects [18, 19, 33, 34]. VC exhibits antioxidant activity by donating its electrons and preventing other compounds from being oxidized, and many laboratory and human studies have established a correlation between VC supplementation and blood pressure reduction [26, 29, 35, 36].

In the present study, VC reduced SBP and DBP in SHRs after four weeks of administration at both a relatively low dose (250 mg/60 kg body weight/day) and a high dose (2000 mg/60 kg body weight/day), and there was no significant difference among the VC groups treated at four different doses of VC (Figure 2). Although the SBP of the VC 2000 group was significantly lower than the VC 500 SHR group in the sixth week, this difference was not shown in other weeks. Therefore, the effect of VC on blood pressure reduction seems to be not dose-dependent in this study. Therefore, the lower concentrations of 250 mg and 500 mg/60 kg were selected, which could obtain similar results with a small amount. In addition, our study was to investigate the effect of the frequency-dependent manner of VC in SHR. The groups treated with VC twice a day (VC 125 *∗* 2 and VC 250 *∗* 2 groups) displayed a decreased SBP after the second week compared with the SHR group. Moreover, the SBP of twice-a-day groups was significantly lower than once a day groups in the second week (VC 125 *∗* 2 vs. VC 250), fourth week (VC 125 *∗* 2 vs. VC 250), sixth week (VC 250 *∗* 2 vs. VC 500), and eighth week (VC 250 *∗* 2 vs. VC 500). Further, in the sixth and eighth weeks, the SBP of twice-a-day groups was similar to that of the normotensive WKY group, whereas the SBP of once-a-day groups was significantly higher than that of the WKY group (Figure 3). Collectively, these results suggest that the effect of VC on blood pressure reduction in SHRs is more effective with increased administration frequency than with increased dose.

As shown in Figure 4(b), the production of ACE I protein in the SHRs was higher than in the WKY group, and VC administration significantly reduced ACE I protein production in SHRs. In addition, twice-a-day groups showed a greater decrease in ACE I protein production than once-a-day groups. There was no significant difference in the production of angiotensinogen among the groups, but groups administered with VC showed a tendency to have increased angiotensinogen production compared with the SHR group (Figure 4(a)). A previous study suggested that VC diminishes Ang II-induced attenuation of endothelium-dependent vasodilation by increasing the production of superoxide anion [9]. Wang et al. [28] showed that prenatal VC treatment decreases mRNA and protein levels of ACE I following prenatal lipopolysaccharide exposure. VC was previously reported to decrease the binding affinity of the angiotensin II receptor type I for Ang II [37]. Based on our findings and previous studies, we suggest that VC attenuates ACE I production as well as the action of its product, Ang II, which may have been responsible for the reduced blood pressure. In addition, the difference of blood pressure between twice-a-day and once-a-day groups might be attributed to differences in ACE I production.

As shown in Figure 5, both eNOS protein production and total NOx concentration in the SHR group were higher than in the WKY group. Interestingly, the total NOx concentration in the blood serum was significantly decreased by VC administration, despite the tendency of these animals to exhibit increased eNOS production levels (Figure 5). Previous studies suggest that the expression of eNOS decreases in SHRs [38]. Our study showed that the level of eNOS increased in SHRs compared to the WKY group but, in the VC treatment groups, the eNOS was increased compared to the SHR group. In other studies, SHRs exhibited an increase in NOx or nitrate production in blood plasma compared with WKY rats [39, 40]. In order to induce smooth muscle relaxation, NO must be transported from endothelial cells to the vascular smooth muscle cells. Figueroa et al. [41] demonstrated that the release of NO from endothelial cells to smooth muscle cells requires connexin (Cx)-based channels. It has been reported that expressions of Cx37, Cx40, and Cx43 were reduced in the vascular endothelial cells of SHRs compared with WKY rats [42–44]. Further, Edwards et al. [45] have reported that VC prevents the effect of Cx peptides that attenuate the transmission of NO from endothelial cells to smooth muscle cells against Cx37, Cx40, and Cx43 as well as blocks the effect of 2-aminoethoxydiphenyl borate, which is a putative gap junction blocker. Therefore, reduced NOx concentration in the serum of SHRs through VC administration suggests that VC enhances the bioavailability of produced NO in the vascular smooth muscle cells and lowers blood pressure by blood vessel relaxation.

## 5. Conclusions

In this study, our results suggest that the cardiovascular system of the SHR reacts to high blood pressure by upregulation of the NO pathway by a protein-induction mechanism, leading to an increase in the production of NO. Taken together, the results in this study suggest that VC produces an antihypertensive effect by modulating the RAS and eNOS-NO pathway, which may be more effective by increasing the frequency of VC administration than the concentration of VC.

## Data Availability

The data used to support the findings of this study are available from the corresponding author upon request.

## Supplementary Materials

Supplementary Materials Data on the blood pressure changes used to support the findings of our research are available in Supplementary Figures S1-S2. Supplementary Figure S1: the blood change of dose of vitamin C administration. Supplementary Figure S2: the blood change of vitamin C administration frequency. Moreover, we have provided all the original uncropped ECL images indicating the proteins we have probed for in supplementary Figure S3.
