# Supplementary material for: Vitamin C Lowers Blood Pressure in Spontaneously Hypertensive Rats by Targeting Angiotensin-Converting Enzyme I Production in a Frequency-Dependent Manner
Source: Evid Based Complement Alternat Med. 2022 Jul 8;2022:9095857. doi: 10.1155/2022/9095857 (PMC9286971; doi:10.1155/2022/9095857)
Supplement: Supplementary Materials — Data on the blood pressure changes used to support the findings of our research are available in Supplementary Figures S1-S2. Supplementary Figure S1: the blood change of dose of vitamin C administration. Supplementary Figure S2: the blood change of vitamin C administration frequency. Moreover, we have provided all the original uncropped ECL images indicating the proteins we have probed for in supplementary Figure S3. [file 9095857.f1.docx]

**Supplementary Materials**

**
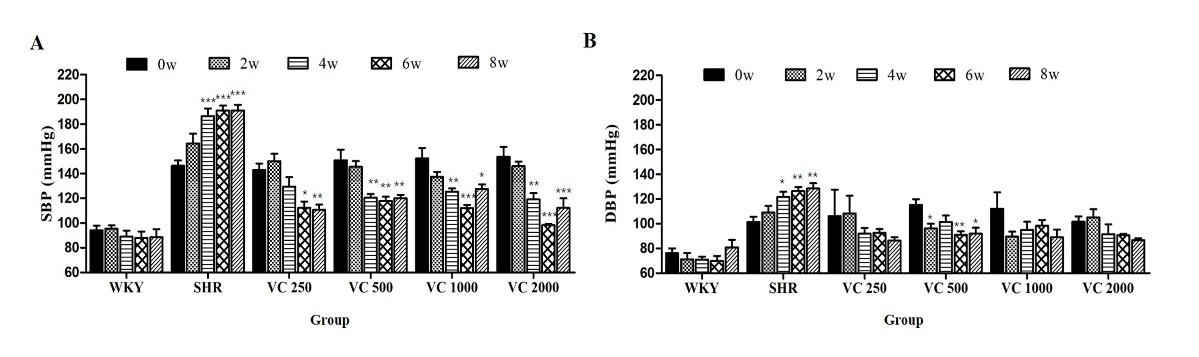
Figure S1.** Effects of dose of vitamin C (VC) administration on (**A**) systolic blood pressure (SBP) and (**B**) diastolic blood pressure (DBP) of the Wistar Kyoto rat (WKY) group (normotensive group), spontaneously hypertensive rat (SHR) group (control group), and VC-administered SHR groups (treatment groups). WKY group (tap water at 9 a.m. and 9 p.m.), SHR group (tap water at 9 a.m. and 9 p.m.), and VC-administered SHR groups (250, 500, 1000, and 2000 mg/60 kg body weight/day of VC; VC at 9 a.m. and tap water at 9 p.m.) over 8 weeks. Each group consists of five rats. Tukey’s honestly significant difference (HSD) test: **p* < 0.05, ***p* < 0.01, ****p* < 0.001 vs. 0 week for each group.

**
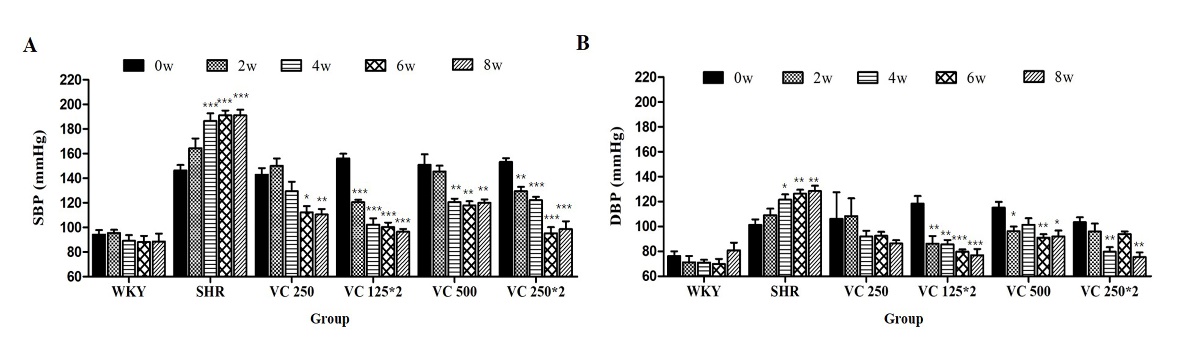
Figure S2.** Effects of VC administration frequency on (**A**) SBP and (**B**) DBP of the WKY group (normotensive group), SHR group (control group), and VC-administered SHR groups (treatment groups)**.** WKY group (tap water at 9 a.m. and 9 p.m.), SHR group (tap water at 9 a.m. and 9 p.m.), and VC-administered SHR groups (250 and 500 mg/60 kg body weight/day of VC; VC at 9 a.m. and tap water at 9 p.m.; 125*2 and 250*2 mg/60 kg body weight/day of VC; VC at 9 a.m. and 9 p.m.) over 8 weeks. Each group consists of five rats. Tukey’s HSD test: **p* < 0.05, ***p* < 0.01, ****p* < 0.001 vs. 0 week for each group.

**
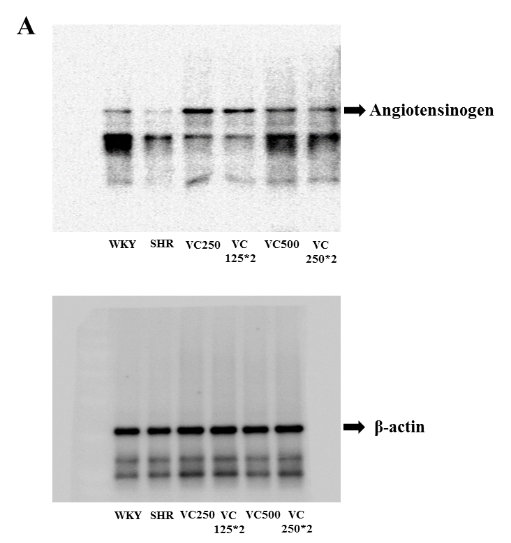

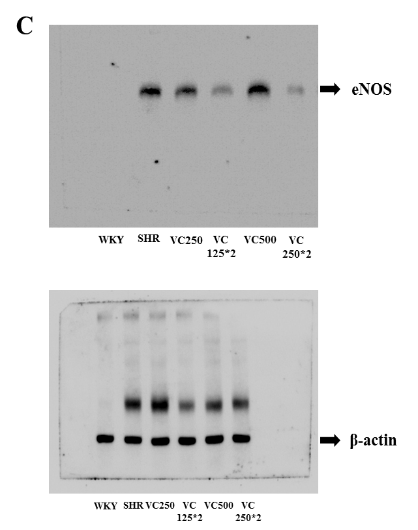

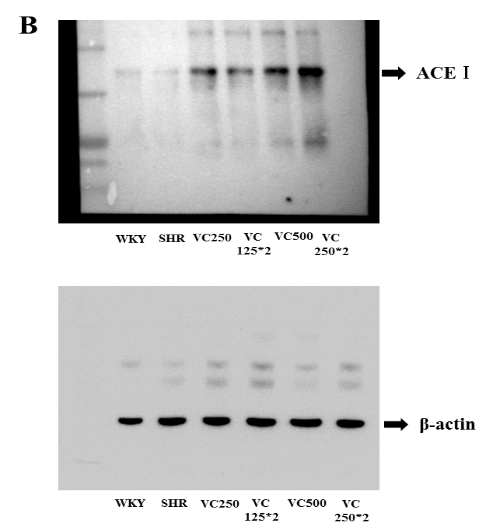
**

**Figure S3.** Original uncropped images indicating (A) Angiotensinogen and β-actin, (B) angiotensin-converting enzyme (ACE) I and β-actin, (C) endothelial nitric oxide synthase (eNOS) and β-actin for representative Western blots used in Figure 4 and 5A of the manuscript.
